# Assessing DRG cost accounting with respect to resource allocation and tariff calculation: the case of Germany

**DOI:** 10.1186/2191-1991-2-15

**Published:** 2012-08-30

**Authors:** Matthias Vogl

**Affiliations:** 1 Helmholtz Zentrum München, German Research Center for Environmental Health, Institute of Health Economics and Health Care Management, Ingolstädter Landstraße 1, 85764 , Neuherberg, Germany; 2 Ludwig-Maximilians-Universität München, Munich School of Management, Institute of Health Economics and Health Care Management & Munich Center of Health Sciences, Ludwigstraße 28, 80539 , München, Germany

**Keywords:** Hospital reimbursement, Hospital costs, Accounting, Resource allocation, Prospective payment system, Diagnosis related groups

## Abstract

The purpose of this paper is to analyze the German diagnosis related groups (G-DRG) cost accounting scheme by assessing its resource allocation at hospital level and its tariff calculation at national level. *First,* the paper reviews and assesses the three steps in the G-DRG resource allocation scheme at hospital level: (1) the groundwork; (2) cost-center accounting; and (3) patient-level costing. *Second,* the paper reviews and assesses the three steps in G-DRG national tariff calculation: (1) plausibility checks; (2) inlier calculation; and (3) the “one hospital” approach. The assessment is based on the two main goals of G-DRG introduction: improving transparency and efficiency. A further empirical assessment attests high costing quality. The G-DRG cost accounting scheme shows high system quality in resource allocation at hospital level, with limitations concerning a managerially relevant full cost approach and limitations in terms of advanced activity-based costing at patient-level. However, the scheme has serious flaws in national tariff calculation: inlier calculation is normative, and the “one hospital” model causes cost bias, adjustment and representativeness issues. The G-DRG system was designed for reimbursement calculation, but developed to a standard with strategic management implications, generalized by the idea of adapting a hospital’s cost structures to DRG revenues. This combination causes problems in actual hospital financing, although resource allocation is advanced at hospital level.

## Background

The German diagnosis related groups system (G-DRG system) was based on the Australian Refined DRG system (AR-DRG system), and was introduced mandatorily in 2004 for the whole acute inpatient sector except psychiatric and psychosomatic treatment. Besides reimbursement calculation, DRGs are now important for budgeting and cost control in hospital management [1]. Thus, an exact full cost approach for the complete cycle of care for a medical condition should be the goal of the costing processes [2]. The G-DRG system and its standardized cost accounting scheme are used for national reimbursement calculation and influence strategic management decisions in hospitals. The demographic characteristics, diagnoses, and clinical interventions of a patient define medically and economically homogeneous groups: DRGs. These groups are allocated by certified grouping software, getting their parameter settings from the Institute for the Hospital Remuneration System (InEK), which is the German calculation authority responsible for reimbursement rates. The accuracy of reimbursement and the practical relevance of the standardized cost accounting scheme are dependent on precise resource allocation and an unbiased, representative tariff calculation by the InEK. Yet resource allocation at hospital level and tariff calculation at national level for German inpatient relative prices (case-mix) are rarely reviewed and assessed in the literature. Although many arguments are already scattered in the literature, an overall assessment of the G-DRG cost accounting scheme is still lacking.

G-DRGs do not cover capital costs and are supplemented by (partly self-negotiated) reimbursement for special treatments or technological innovation not yet in the DRG catalog [3,4] and by DRGs without case weights (ca. 40) [5]. These DRGs are reimbursed separately for each hospital as a result of inhomogeneity of care, resulting in high variance in costs or a very small calculation base [6]. Hospital costs can be calculated according to the InEK calculation scheme at different aggregation levels (hospital/department/DRG group/DRG/case) to compare the refinanced costs in each cost category/cost-center segment. This great detail at different aggregation levels, up to the patient-level, makes this scheme very valuable and flexible for strategic management decisions. All German hospitals have the possibility to participate in the calculation process for G-DRG reimbursement rates with their case cost data. The cost weights of reimbursement rates for DRGs are recalculated each year by the InEK on the basis of hospitals providing the previous year’s cost accounting data in a default scheme (263 or 16% of all German hospitals in 2009). Base rates that form the actual reimbursement rates by multiplying them by the calculated DRG cost weights are the subject of negotiation in each state. Besides the development of the grouping software, the InEK cost accounting scheme is *the* driver for German inpatient relative prices (case-mix). This de facto accounting standard not only allows the calculation of reimbursement rates, it can also be used for benchmarking, strategic planning, and to compare costs and revenues in calculating and non-calculating hospitals [7]. Besides its budget relevance, the G-DRG system has also started to be used as a pricing system [8]. The case-mix and the case-mix index contribute to the most important financial ratios in hospital management besides liquidity, cash flow, and contribution margin [9]. Papers published so far have analyzed the G-DRG system in a comprehensive way [10,11]. An explicit analysis of the costing side and whether G-DRG cost accounting meets the two main reasons for the introduction of the G-DRG scheme (transparency and efficiency) are not part of the literature yet. Besides improving efficiency and transparency, the G-DRG scheme has been suspected of compromising on quality of care (e.g., early discharge or cream-skimming) and documentation of service delivery (e.g., up-coding) [12]. As cost accounting has no direct influence on the quality of care, this aspect is not analyzed in this paper. Although it is difficult to prove this influence, as these systems (per-diem charges and case fees) did not exist in parallel, the latest official DRG-impact-evaluation did not find a negative impact on the quality of care, but some documentation issues during the years of its introduction [13].

## Methods

**Figure 1:**
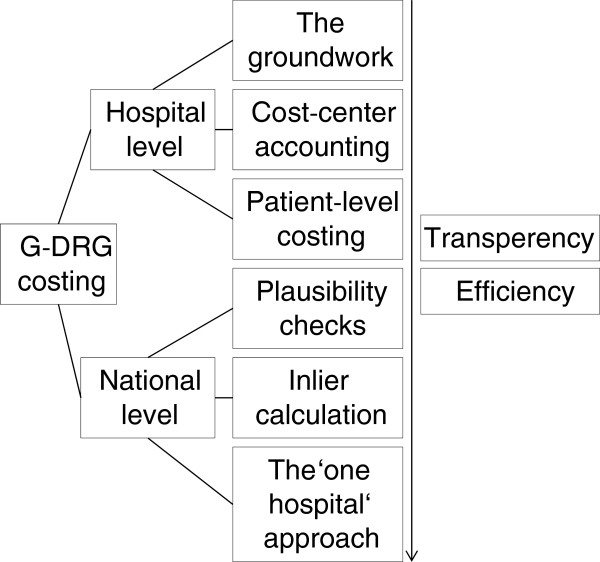
Goals in the G-DRG costing process.

An executive summary is given of the InEK cost accounting scheme at hospital level and on the determination of reimbursement rates at national level, to show the course of action in calculating German inpatient relative prices. To measure improvements since its introduction, and to enable an objective international comparison and classification, an empirical approach is used, measuring cost homogeneity within DRGs. The three steps of resource allocation at hospital level and the three steps of tariff calculation at national level are assessed, with reference to the two main goals of DRG introduction: improving efficiency and improving transparency [14]. First, the paper reviews and assesses the three steps in G-DRG resource allocation scheme at hospital level: (1) the groundwork; (2) cost-center accounting; and (3) patient-level costing. Second, the paper reviews and assesses the three steps of G-DRG national tariff calculation: (1) plausibility checks; (2) inlier calculation; and (3) the “one hospital” approach (see Figure 1). 

## Resource allocation at hospital level

### The groundwork

This section demonstrates the course of action in the G-DRG cost accounting system to allow a detailed assessment of the ability of the scheme to accommodate differentiated resource allocation and precise cost assessment. Costs of outpatient services and psychiatric services are calculated separately or are completely excluded from the calculation (not only in the G-DRG scheme) [15-18]. Currently, new, separate systems for psychiatric services and outpatient cases are being developed within G-DRG costing: cost accounting as presented in the following is adapted in principle to psychiatric services, but with a greater focus on length of stay concerning calculation and reimbursement [19]. According to the InEK handbook for calculation, participating hospitals have to build up their case costs and case calculation. A detailed description of the following cost accounting scheme is given in the InEK calculation manual for calculating case costs, version 3.0 [15]. Participating hospitals have to meet basic costing modalities: cost allocation on each inpatient case relies on a full cost approach using actual costs. This full cost approach refers to DRG-relevant costs only. The audited annual accounts build the cost frame and the calendar year defines the calculation period. Direct costs (drugs, blood, implants, etc.) and overhead costs are distinguished for a subsequent allocation. Costs of general indirect cost-centers with patient activity are allocated to the respective direct cost-centers. In particular, labor costs (medical staff, nursing staff, med.-technical staff, and ancillary staff) have to be allocated to cost-centers according to actual utilization.

**Figure 2:**
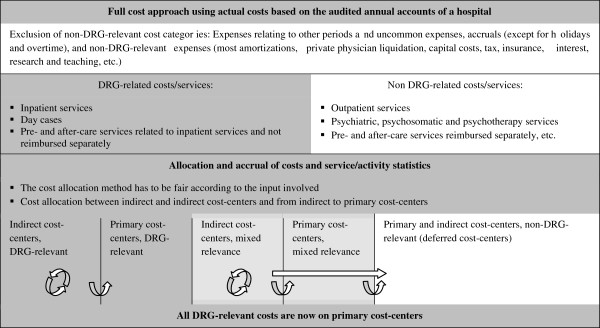
** The cost accounting scheme in the G-DRG system, Source.**[15].

**Figure 3:**
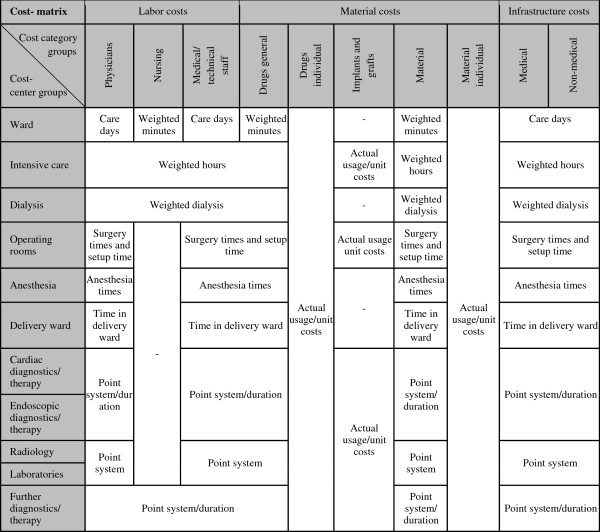
** The cost-matrix for every case, Source**[15]. *Notes:* Cost-centers and cost categories are merged to the cost-center groups and cost category groups shown in the cost-matrix. Costs are allocated to cases according to the key cost drivers for cost modules shown in the cost-matrix.

Only DRG-relevant costs (necessary for DRG-related utilization: medical treatment, care, drugs, cures, therapeutic appliance, and board and lodging) remain in the calculation. Non-DRG-relevant cost categories and cost-centers have to be eliminated from the calculation or, in the case of partial relevance, split according to cost-center-specific, DRG-relevant resource utilization. Thus, an exclusion of costs at the highest possible level of aggregation is claimed (full cost approach based on DRG-relevance). The most important non-DRG-relevant costs arise for ambulatory care, research and teaching, psychiatric care, extraordinary expenses, and expenses not relating to the calculation period. Non DRG relevant cost categories are accruals (except for holidays and overtime), most amortizations, private physician liquidation, capital costs, tax, insurance, interest, etc. All cost-centers have to be classified to primary cost-centers (service to patient) or indirect cost-centers (medical and non-medical infrastructure). Figures 2 and 3 explain the groundwork and the subsequent cost-center and patient-level costing.

### Cost-center accounting

The basic initial point in cost-center accounting, defined by law for all German hospitals, is a defined set of cost-centers and cost categories. Thus, all German hospitals comply with the prerequisites of cost-center accounting. To get closer to the cost-matrix scheme claimed by the InEK, cost categories have to be merged into groups (see the cost-matrix in Figure 3), in which the cost categories of costs on indirect cost-centers switch to medical and non-medical infrastructure. The use of internal cost allocation methods is restricted to accurate ones that also take the service exchange between indirect cost-centers into account, such as the iterative method of assessment [20,21]. Compensation keys (cost drivers for the allocation of costs between cost centers, e.g., cases for administrative costs or floor area for maintenance costs) have to follow the method of causation. Thus, costs on indirect cost-centers have to be allocated to primary cost-centers, and excluded in case of non-DRG relevance. Costs on primary cost-centers might have to be relocated to the primary cost-center they accrue in. After the internal cost allocation, non-DRG-relevant parts of primary cost-centers have to be excluded from the calculation according to the key cost driver (service/activity statistics) of the primary cost-center.

### Patient-level costing

A uniform cost-matrix for every calculated case has to be generated according to the cost-matrix in Figure 3. Cost-centers are allocated to the cost-center groups. Direct costs, which are compulsory for drugs, implants, transplants, blood products, costly external services, etc., are allocated on a patient-basis, according to the documented utilization. Sometimes, an allocation based on a clinical distribution model is allowed. This is a consistent allocation of costs on cases with the same services or procedures related to the drug or medical device. Overhead costs and costs on primary cost-centers (that are not documented as patient based) are allocated based on the key cost driver (weighted or un-weighted) for each cost module in Figure 3. A calculation of allocation bases that is un-weighted is not allowed in most cases. This is a uniform distribution of costs on cases without case-related key cost drivers (service/activity statistics). In most cost modules, a weighted calculation of allocation bases is claimed. This makes case-related key cost drivers for each cost-center necessary to distribute costs to cases. Examples of compulsory key cost drivers and service/activity statistics, as in the cost-matrix in Figure 3, are days on the ward, differentiated operating room minutes, point systems for diagnostics, radiology, and laboratory services for the allocation of medical staff costs. The calculation of allocation bases has to be performed at the level of primary cost-centers, not at the level of cost-center groups. This is necessary to get an exact allocation, based on the actual services and activities in the cost-center/category, not in the less accurate cost-center/category groups.

## Tariff calculation at a national level

### Plausibility checks

After case-cost calculation at hospital level, tariff calculation at national level is the second step on the way to understanding the development of G-DRG reimbursement rates and hospital management decisions. The InEK uses plausibility and conformity checks on patient-level data to decide in each case whether costs are calculated according to the guidelines, and whether the case can be included in the nationwide DRG-calculation [6]. Therefore, participating hospitals provide case-cost data (a cost-matrix for every case), clinical case data (diagnoses, operations, and procedures, gender, age, etc.), additional service data (further service/activity information, e.g., operating room minutes, methods of allocation used, etc.), and a control total for costing based on the audited annual accounts of the hospital. These data are first checked for technical and formal errors. Then a medical check (to test accordance with coding standards), an economic check (content based for the hospital, cost-center groups, and cases), and a check combining both (e.g., interdependence of costs and services/procedures) are used to detect possible documentation or calculation errors and to allow for correction. Further, the allocation method and the additional service data are tested in overall conformity checks, such as a missing correlation of costs and service/activity statistics or wrong allocation methods.

Case costs in cost modules have to relate to case activity, given default band widths. And the relative costs of cost-modules also have to be in default band widths to each other. Participants are informed about errors or possible errors, and have to revise or explain in several rounds. Through this process, the quality of the case costs should be enhanced. All these checks are performed at single patient-level. Case costs of accepted cases from the hospital are used for calculation if unaccepted cases do not exceed a certain percentage, and if the hospital passes overall conformity checks, analyzing whether the requested calculation methods and processes are met. Plausibility and conformity checks have become more severe in recent years. This has included shrinking the acceptable band-width in cost modules, more queries that result in more detected calculation errors, and the introduction of the mandatory delivery of additional service data, such as requests for very detailed operating room statistics. The overall percentage of cases with calculation errors allowed decreased. Because of the additional service data, conformity checks could have intensified in recent years. Possible reasons for the decreased number of calculating hospitals in 2008 are the introduction of more severe plausibility checks or the high number of excluded hospitals in the previous year, which lowered the motivation to participate and reduced the percentage of cases surviving plausibility checks (see Table 1).

**Table 1:** **Calculation activities from 2004 to 2011, Source**[6,22,25-31]

*G-DRG- system	**Number of DRGs**	**Number of specialized additional services**	**Number of calculating hospitals**	**Number of calculating hospitals excluded**	**Calculating hospitals (%)**	**Cases after plausibility check**	**% of cases surviving plausibility checks**	**Fraction of all German inpatient cases (%)**
2004	824	26	144	n/a	8.1	2,395,410	84.8	13.5
2005	878	71	148	n/a	8.5	2,283,874	83.4	12.8
2006	954	82	214	n/a	12.0	2,851,819	80.7	16.1
2007	1,082	105	263	38	14.9	2,863,115	67.5	16.3
2008	1,137	115	249	28	14.3	2,811,669	72.1	15.8
2009	1,192	127	251	33	14.5	3,075,378	70.3	16.9
2010	1,200	143	253	28	14.8	3,257,497	71.8	17.5
2011	1,194	146	263	16	15.9	3,501,515	71.9	18.5

*calculation data always from previous year (e.g., G-DRG system 2011 based on 2010 data).

### Inlier calculation

The DRG system should group cases medically and cost homogeneously. Therefore, only “inliers” – cases within a certain standard length of stay (LOS) period, defined by a lower and an upper trim point – become part of the calculation for the standard reimbursement rates. Cases below or above the standard LOS attract deductions or additional reimbursement to embrace the changed cost situation [22]. Cases relocated to another hospital before their mean LOS also attract deductions. By adding the fees, an effective cost weight is generated for every case. Upper and lower trim points, and the related deductions or additional reimbursement, are derived normatively. For example, the lower trim point is one third of the average LOS but has a minimum of 2 days. Related formulas (see [11,22]) are not presented here, as they do not contribute to the assessment of the scheme according to its main goals: transparency and efficiency.

### The “one hospital” approach

The calculation at national level follows the “one hospital” approach, meaning that DRGs of all calculating hospitals are dealt with as though coming from one hospital. To determine the national cost weight of a specific DRG, the costs of all accepted inliers within the respective DRG are divided by the allocation base. Before 2006, the allocation base was the weighted arithmetic mean of the costs of all “inliers” meaning that the average case weight of all inliers was 1 [22]. Since 2006, the allocation base has been calculated in a way that keeps the sum of all case weights in Germany constant and also includes “outliers” to make it comparable over time [23]. Only changes in the underlying dataset, such as adding or removing DRGs, or changes in supplementary fees affecting DRGs can change the German case weight sum of 2006 as a constant basis. Owing to the development of the system, an overall technical (methodic/calculative) effect was induced that influenced case weights, but had nothing to do with the development of the DRG classification. A so-called liquidity effect could arise as a result of individual, retrospective negotiations for the base rate of the hospital, as the individual base rate could only react as a delay to the technical effect. To reduce this effect and to make the allocation base comparable with previous years, the calculation of the allocation base had to change. Reimbursement fees are then calculated by multiplying the calculated case weights by the – in every state – separately and recently negotiated base rate. The negotiation of the base rate is influenced by factors such as inflation, regional price index, and collective wage agreements. This base rate was hospital specific (budget neutral) at the time of G-DRG introduction from 2003 to 2004. From 2005 to 2009 hospital-specific base rates converged to a state-wide base rate and, from 2010 to 2014, the state-wide base rates should converge to a nationwide base rate in a corridor of 2.5% above and 1.25% below this nationwide base rate [10,24]. So the “one hospital” approach already reached by calculating DRG cost weights is further established with base rates until 2014.

**Figure 4:**
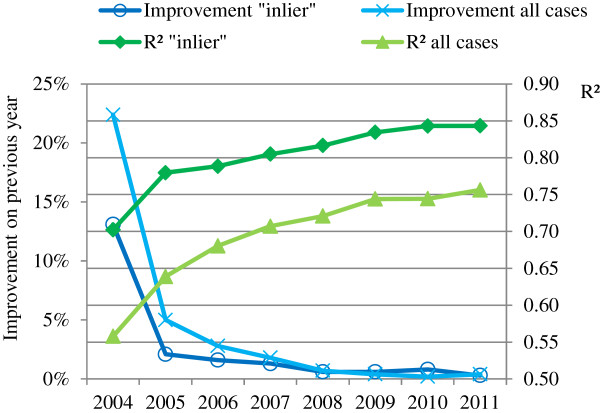
*** R²* over time (based on each year’s G-DRG system), Source**[6,22,25-30].

An empirical approach to quantify improvements in G-DRG tariff calculation is to analyze the reduction in variance of costs, which the G-DRG scheme approaches by accurate G-DRG tariff calculation. An analysis of the coefficient of determination *R*^*2*^ over time (G-DRG versions 2004–2011) shows the explained part of the statistical spread of costs resulting from DRG classification. On account of the allocation of case costs to nearly 1,200 DRGs, similar costs for cases in high-volume standardized treatment DRGs and additive components for special treatments, medical products, etc., the explained part of inlier case-costs in the G-DRG system is already very high. Over time, the extent of variance reduction is measured. Figure 4 shows the explained part of costs with an *R*^*2*^ > 0.8 for inlier cases (*R*^*2*^ > 0.7 with outliers included) in recent years. However, a ceiling effect is noticeable [6,22,25-31]. The contribution of the increased number of DRGs to a rising *R*^*2*^ is negligible, with an adjusted *R*^*2*^ always very close to *R*^*2*^[6,32]. This high declared portion of variance is not unusual, as the Australian AR-DRG-system from 1998 already showed a reduction in variance (RIV) of 68%, and the English PbR costing system also showed a RIV of over 80% in 2007–2008 [33,34]. 

**Figure 5:**
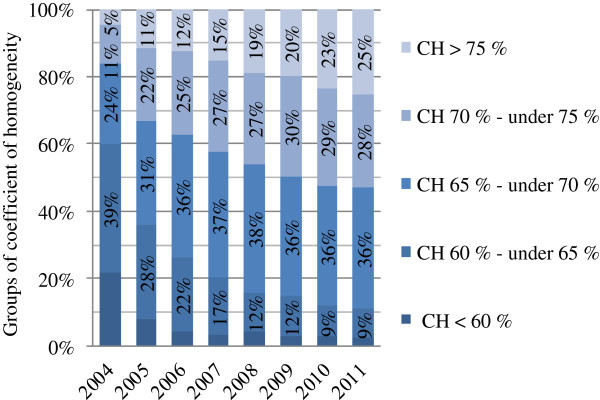
** The Coefficient of Homogeneity over time (based on each year’s G-DRG system), Source**[6,22,25-30].

Cost homogeneity of DRGs can be analyzed further using the coefficient of homogeneity *CH = 1/(1+ σ/μ)*, a measure of the statistical spread or the uniformity within a certain group. It derives from the coefficient of variation *σ/μ* (*σ* = standard deviation, *μ* = mean). A *CH* of 1 indicates full homogeneity, whereas a *CH* close to 0 indicates no homogeneity (see formula). Figure 5 splits the coefficient of homogeneity of costs into groups and shows the percentage of DRGs in these groups for the G-DRG versions 2004–2011 [6,22,25-30]. The coefficient of homogeneity of costs and the coefficient of determination *R*^*2*^ from 2004 to 2011 confirm a developing G-DRG cost accounting system with improving resource allocation and cost assessment, with a low unexplained cost spread within DRGs. 

Calculation requirements for participating hospitals have increased greatly in the last 8 years. Internal cost allocation methods were very rudimentary at the beginning, especially concerning (non-)medical infrastructure [11,35]. In former versions of the cost accounting scheme, infrastructure costs did not have to be distributed completely to cost-center categories, resulting in an uncertain allocation of large parts of overall costs. Accepted key cost drivers to allocate on cases are more precise now, and less choice is offered. Since 2006, advanced internal cost allocation methods such as the iterative method have been made compulsory, and compensation keys for the allocation are default states [15]. Table 1 gives an overview of calculation activities from 2004 to 2011 [6,22,25-30].

## Discussion

### Resource allocation at hospital level: the groundwork

G-DRG costing excludes the costs of teaching and research, as these have a wide range among hospitals and not all hospitals have teaching and research units. Accruals, most amortizations, private physician liquidation, capital costs, tax, insurance, and interest are still not part of the tariff calculation, although DRG relevant. This facilitates benchmarking, but contrasts a useful full cost approach and managerial relevance. Although the separation of capital costs (historically reimbursed separately [3]) increases national comparability, it does not respect different capital cost requirements resulting from the case-mix of a hospital. Thus, an inclusion in G-DRG costing, as currently developed, is desirable. As it does not seem applicable to completely reduce reimbursement to DRGs and keep the number of DRGs manageable, additional fees for highly specialized services in various degrees (e.g., for expensive drugs and treatment or intensive care) are suitable. This allows a fair reimbursement of highly specialized or expensive services. The exclusion of DRG-relevant costs in G-DRG costing limits efficiency, as reimbursement is then partially not based on actual costs. Transparency is limited, as the sources and magnitude of capital costs cannot be compared among hospitals. Systematic, software-based calculation inequalities have to be incorporated when reflecting a hospital’s DRG costs. Currently, unlike in Australia, hospitals are free to decide on accounting and hospital management software. A certification process such as that applied for the DRG grouping software does not exist for cost accounting programs referring to the InEK calculation [36]. Thus, transparency is further limited.

### Resource allocation at hospital level: cost-center accounting

The breakdown of cost-centers into DRG-relevant and non DRG-relevant costs is sometimes hard to establish, e.g., because of non-separated working time accounts for inpatient and ambulant patients. Other difficulties in cost-center accounting are resolved well: internal allocation methods such as the iterative method are advanced; compensation keys are appropriate; and the cost-center/cost category breakdown is detailed. Key cost drivers at patient level are allocated on the basis of cost-centers/categories, not on the basis of cost-center/category groups, improving accuracy. The hospital costing literature gives no hints as to how this stage might be improved. Thus, transparency and efficiency are given at cost-center level.

### Resource allocation at hospital level: patient-level costing

Participation rates in the InEK calculation scheme are around 16%, as hospitals often do not have the cost accounting prerequisites to calculate at the patient-level [37]. Feeder systems in cost-centers such as radiology cannot provide the key cost drivers necessary (see Figure 3) to participate in the calculation. A future cost-modeling approach for non-calculating hospitals might support representativeness without downgrading the accounting standards or making participation in the calculation mandatory – which is hardly achievable because of the costly accounting prerequisites that participation in reimbursement calculation requires. Providing public service weights (as in Australia) for hospitals with less advanced accounting systems or accepting less advanced key cost drivers in some cost modules are a practical solutions to this issue [36]. Graded calculation methods, representing the technical accounting capabilities of a hospital, as in the English PbR system, are already well-proven [38]. Still, a high percentage of cost distribution is based on LOS, and especially for medical DRGs (conservative therapy), the fraction of directly case-related costs is low compared with operative DRGs. Although key cost drivers allocated to patients improved (see Figure 3), a sophisticated solution has not been implemented yet. The use of key cost drivers based on old and imprecise point systems, originally developed for physician reimbursement, results in imprecise capacity and resource planning (e.g., operating room or staff costs for DRGs). Economically sound decisions become limited. The use of the G-DRG system based on InEK calculation as a pricing system and not only as a budgeting instrument is critical against this background [8]. To improve costing at the patient-level, Kaplan & Porter (2011) introduced time-driven activity-based costing (TDABC) in the hospital [37,39]. This allocation method defines nearly all costs as variable and includes time as a key cost driver in a more detailed implementation, such as duration of clinician visits as an example of direct costs or the duration of laboratory tests as an example of indirect costs. In some cost modules, such as physicians in the operating room, TDABC is already implemented in the InEK costing standard. The more detailed the calculations are, the more precisely and efficiently management can act; and management decisions become transparent.

Overall, transparency and efficiency in cost accounting have improved since the introduction of the InEK costing scheme. The calculation manual for the calculation of case costs is in its third version now and has increased costing standards greatly since its introduction. Its implementation is enforced by improved and more rigorous plausibility checks. At the hospital level, the learning effect, paired with developing cost accounting software and documentation requirements, has kept up with increased calculation requirements. After an adaptation phase, the coding of diagnoses and operations and procedures has remained relatively stable [13]. Hospitals try to adapt treatments to the DRG-system (reimbursement) by implementing clinical pathways, also oriented on the InEK calculation scheme [40], promising higher efficiency [41] and increased transparency through better documentation of the course of action.

### Tariff calculation at national level: plausibility checks

The claimed advanced cost accounting methods at hospital level contain risks for tariff calculation. The control for tight band-width in cost modules and further plausibility checks can lead to the exclusion of cases from the calculation, even if costs are calculated correctly. If the costs of a case in a single cost module are not in a target corridor based on previous years, the case might be excluded [6]. As these corridors are not published, the actual influence and control mechanism of band-widths cannot be defined. Future research is needed to analyze bias caused by band-width control. Too closely meshed plausibility checks might lead not only to data quality improvements, but also to an undesirable trimming of the calculation on InEK plausibility checks. To achieve high data quality, plausibility checks are both a blessing and a curse. On the one hand, they enforce improved cost accounting, resulting in improved data quality. On the other hand, they might push hospitals into default band-widths, possibly not representing the hospitals’ actual cost structure. To improve participation rates, fees for every case passing plausibility checks were introduced, promoting the focus on plausibility checks and questioning the “one hospital” approach, as further bias is introduced. The efficiency goal of DRG introduction is therefore slightly transgressed, although plausibility checks contribute to efficiency overall. Transparency is improved, as calculation errors are reported on a patient basis in every round. However, the background on band-width calculation should be reported better and the kind of calculation errors should be made public (e.g., the top 100 calculation errors) to let hospitals benefit from early avoidance of these calculation errors in future calculations. To further reduce the rate of cancelled data, an automated system should be introduced for reporting error explanations on a case basis, enabling the InEK to produce statistics from the hospitals’ point of view on specialized calculation errors.

### Tariff calculation at national level: inlier calculation

That the calculation of length of stay (LOS) thresholds is highly important concerning incentives for providers has been shown in detail [42], also implying that coding issues can be reactions to inlier calculation [43]. Still, normatively derived upper and lower LOS thresholds imply systematic failures and possibly underfinancing [44,45]. The deductions from reimbursement rates due to short stay are not calculated based on cost accounting data [44,45]. They follow a “main effort concept,” including deductions for services not part of the main effort [45]. Therefore, other countries such as England do not use lower trim points [46]. Upper trim points were introduced to lower the risk of the hospital in complicated cases; however, they also suffer from their normative derivation. A non-normative costing-based calculation as in the U.S. (“cost outliers” [47]) or a specific case-mix for each care day (Victoria, Australia [48]) might be a solution for upper and lower trim points, also embracing rare DRGs. Otherwise, supererogation can lead to a reduced effective reimbursement rate [44]. This partial “system failure” can have dramatic costing impacts in some cases, as outliers are not in the minority. The 2011 G-DRG system had an average of 22.3% outliers (standard deviation 12.7%, ranging from 0% to 83.2% outliers within DRGs) [49]. Inadequate trim point calculation opens up the discussion of a greater DRG differentiation, implying unintended single case reimbursement and less practicability in the grade of differentiation [13]. That a higher grade of differentiation might not improve welfare has already been shown in econometric models [50]. Still, consistent outliers can be a sign of the need for further DRG differentiation. Expanding additive components in the DRG calculation, as currently exercised, or outlier-calculation based on costs might partly resolve this issue and increase economic homogeneity [32]. As the generation of outliers is a necessity to reduce the risk for providers and to create medically and economically homogeneous groups (*R*^*2*^ is about 0.1 lower for all cases compared with inliers; see Figure 4), a focus should be set on the influence of the accounting system to define outliers. Further, the explained part of the variance is much higher when referring to costs compared with LOS (normative derivation) [34]. By using the different modules in the InEK matrix in combination with the date of cost occurrence for outlier calculation, the less accurate normative derivation could be replaced. Thus, the current system has high transparency through normative derivation, but serious flaws concerning the efficiency of the calculation.

### Tariff calculation at national level: the "one hospital" approach

The “one hospital” approach causes a bias in costs (regional price index, pay scale, etc.), which can be resolved only partly by the negotiated base rate in every state concerning reimbursement. The InEK only adjusts according to wage index/union rates in the regions of former East Germany [6]. There is no adjustment in the reimbursement for geographical variations in case-costs – an obvious disadvantage for high-cost regions. Besides, owing to the different composition of DRG costs (e.g., labor costs, material costs), DRGs are affected by this non-adjustment to different degrees, which might result in a preference for less labor-intensive DRGs in high wage index areas and vice versa. For example average household income in the 16 German states (Länder) varied between € 4,253 and € 2,617 in 2008 [51]. Although a unique base rate calculation and few regional adaptations support competition, they might contradict the care mandate of every German hospital and undermine the security of full health care supply in every region. The convergence phase of the base rate resulting in a future nationwide base rate shows that the “one hospital” approach, supporting competition, is the chosen route.

Further, case weights are always 2 years old when published. The G-DRG reimbursement of 2012 was calculated with the 2011 G-DRG scheme, based on data collected in 2010. The out-of-date issue affects only relative cost-data (case-mix), as base rates are negotiated for every year in every state. But the quality of tariff calculation suffers from out-of-date relative cost-data and especially from insufficient regional cost adaptation. For management implications, non-adjustment in the “one hospital” model is resolved by putting a higher relevance on the relative cost matrix (the case-mix of a case distributed to the cost matrix), which has to be multiplied by a base rate to get the actual cost. For internal management decisions the base rate can then be adapted to the question that has to be answered. For example, the actual costs published by the InEK are not used as a reference for a hospital’s cases, but the relative costs calculated by dividing the InEK cost matrix for a DRG by the allocation base (the InEK calculation base rate, see “Tariff calculation at national level; the “one hospital” approach”).

Another negative aspect of the “one hospital” model is the unadjusted bias that can be induced by the voluntary participation of hospitals, as the choice of hospitals to take part in the calculation can have many different incentives. In the most favorable case, their motivation to take part is image, the fee for every calculated case, or the wish to compare themselves with a nationwide benchmark, and thus the use of the calculation for internal management decisions. In the worst case, the hospital already uses the InEK cost accounting scheme or equivalent systems for internal strategic management decisions and decides on whether the participation might affect its own future reimbursement positively or negatively. This incentive is especially strong when a hospital knows that it delivers a high percentage of overall cases for the calculation of a DRG, or for hospital chains, where the calculation of one hospital affects the reimbursement of others. Hospitals that are already very efficient have a low incentive to reduce their future reimbursement by delivering beneficial cases. They have the overall vicious circle nature of the system in mind when deciding about participation.

The fact that structure, ownership, and size of the calculating hospitals in general do not reflect the German hospital market is the final problem of the current “one hospital” approach [11]. There is an overrepresentation of medium and large hospitals, as small hospitals are possibly not able to achieve the costly, IT and accounting standards required. Concerning ownership, a biased sample can further affect costs, as the incentive to be efficient depends on ownership structure (private for-profit, private non-profit, public). Most of the literature concludes that private for-profit and private non-profit hospitals are less cost efficient than publicly owned hospitals [52-54]. However, the fraction of actual calculation hospitals (ca. 50% private non-profit, 10% private for-profit, 40% public) does not correspond with the fraction of potential calculation hospitals (ca. 42% private non-profit, 25% private for-profit, 33% public) concerning ownership in recent years [55]. Although the fraction of publicly owned calculation hospitals has stayed the same since the introduction of the G-DRG system, the private non-profit fraction has increased by ca. 6% and the private for-profit fraction has decreased by that amount [22,25-30,55]. Hospitals that have an incentive to improve efficiency also have an incentive to participate in G-DRG calculation, as this is the only generalized system to support management with cost accounting. As a result, the calculated costs tend to be higher than the true German mean.

To compare the profitability of calculating hospitals and non-calculating hospitals in further research might verify the impact of DRG calculation on management quality and system dynamics best, although the mentioned limitations concerning incentives, structure, ownership, and size have to be borne in mind and rethought by policymakers. Limitations in representativeness and non-adjustment affect benchmarking and strategic reactions on reimbursement rates concerning the elective case-mix. They increase the insecurity of hospital management on published DRG costs. Hospitals react to changes in the case fees catalog by changing their elective case-mix [48] – a regionally shaped DRG supply situation can develop, and a vicious circle is initiated concerning the motivation for participation. In contrast, the U.S. has a system that adjusts reimbursement rates for urban and rural areas, for a disproportionate share of poor patients, for regional wage levels, and for teaching [56,57]. The English Payment by Results (PbR) -system uses market force factors (MFF) to adjust to the regional cost situation [16,58]. The adoption of adjustment mechanisms as in the PbR system or the U.S. system would be beneficial for the G-DRG scheme concerning incentives to participate; however, it would lead to overall efficiency losses on account of less competition.

The G-DRG cost accounting system helps to organize the elective DRG-portfolio. Only departments/DRGs with a positive perspective are established or developed; the system protects from misdirected investments, but also favors DRGs with a high yield. The comparability and reproducibility resulting from a standardized G-DRG tariff calculation scheme are of great interest. Transparency and efficiency of tariff calculation are seriously transgressed by the non-representative calculation sample and the motivation to participate. One option to adjust for non-representativeness in the long run is to make the participation of hospitals in InEK cost accounting compulsory. Without reducing the quality of cost accounting by forcing hospitals with less advanced costing abilities to participate, the most important step is to introduce a score that is assigned to the cost modules in the matrix, representing the quality of the allocation methodology for that cost module. The English patient-level information and costing system (PLICS) uses such scores to allow for a high participation rate [59]. To motivate hospitals to reach a high score, the current case fees for cases calculated correctly could depend on score size. Hospitals with less advanced methods can also use key cost driver statistics from hospitals using advanced calculations (official relative value units) to distribute their costs on cases. For an overview of the impact of cost accounting modalities on transparency and efficiency, see Table 2. 

**Table 2:** Assessing the G-DRG cost accounting scheme

	**Goals of G-DRG introduction**		
	**Improving efficiency**	**Improving transparency**	**Author’s recommendation**
Resource allocation at hospital level			
The groundwork	Medium standard/improvements necessary	High standard/small improvements possible	Inclusion of *all* DRG-relevant costs
Cost-center accounting	High standard	High standard	-
Patient-level costing	Medium standard/improvements necessary	High standard/small improvements possible	Improving key cost drivers and further introduction of TDABC
Tariff calculation at national level			
Plausibility checks	High standard/small improvements possible	High standard/small improvements possible	Improving transparency on reasons for calculation errors
Inlier calculation	Low standard/improvements necessary	High standard	Combining normative derivation with the cost outlier concept
The “one hospital” approach	Low standard/improvements necessary	Low standard/improvements necessary	Increasing participation by a lower costing standard parallel to the currentstandard, to reduce participation bias

## Conclusions

This paper assesses the major cost accounting steps in their impact on the goals of G-DRG introduction: improving transparency and efficiency. Based on the highly differentiated cost module and patient-based calculating structure, the InEK calculation scheme for DRG costs has become a de facto standard for benchmarking in inpatient cost accounting and management, and seems to be developing dynamically. The empirical approach has quantified improvements in G-DRG tariff calculation. The system offers most of the tools necessary to improve efficiency. Transparency and efficiency are reached in the calculations at hospital level, with few improvements possible at an advanced patient-based level, such as time-driven activity-based costing or a consistent full-cost approach. However, problematic incentives for participation in the calculation can bias the calculated costs; the G-DRG tariff calculation has a representativeness problem. Although having advanced plausibility checks, tariff calculation methods incorporate the actual cost situation better in other countries. Advancing tariff calculation in hospital financing reforms is a necessity to improve efficiency and transparency in health care management in the long run.

The InEK was able to increase the sample of calculating hospitals, supporting high calculation standards concerning resource allocation at hospital level, but facing methodological problems concerning tariff calculation at national level, such as comparability or inlier calculation issues. As tailored to suit market needs, the standardized cost accounting in the G-DRG system leads to more efficient resource use, more efficient provision of capacity, more transparent and efficient cost-and activity control, and greater competition [13,60]. The latest official DRG-impact-evaluation by Fürstenberg et al. (2011) showed that average length of stay was further reduced within the DRG phase, and average overall costs did not grow faster than before G-DRG introduction (2% per year) [13]. The G-DRG system was designed for reimbursement calculation, but has developed to a standard with strategic management implications, generalized by the idea of adapting a hospital’s own cost structures to DRG revenues. This combination causes problems in actual hospital financing, although resource allocation in the costing scheme is advanced. Still, the G-DRG costing scheme is the best starting-point for management decisions and for the implementation of further patient-level costing. With the mentioned limitations, especially concerning tariff calculation at national level, the current G-DRG costing scheme can be considered to be efficient and transparent.

## Abbreviations

CH: Coefficient of homogeneity; DRG: Diagnosis related groups; G-DRG: German diagnosis related groups; InEK: Institute for the Hospital Remuneration System; LOS: Length of stay; PbR: Payment by results; PLICS: Patient level information and costing system; TDABC: Time-driven activity-based costing; RIV: Reduction in variance.

## Competing interests

The author declares that he has no competing interests.
